# Multi-state Structure Prediction of G Protein-Coupled Receptor Proteins via Prompting on AlphaFold

**DOI:** 10.34133/csbj.0179

**Published:** 2026-09-10

**Authors:** Zhigang Sun, Tao Zhang, Kexin Zhang, Anqi Pang, Jiale Yu, Liting Zeng, Sibei Yang, Suwen Zhao, Jie Zheng

**Affiliations:** ^1^ School of Information Science and Technology, ShanghaiTech University, Shanghai, China.; ^2^ School of Life Science and Technology, ShanghaiTech University, Shanghai, China.; ^3^ School of Computer Science and Engineering, Sun Yat-sen University, Guangzhou, China.; ^4^ iHuman Institute, ShanghaiTech University, Shanghai, China.

## Abstract

G protein-coupled receptors (GPCRs), an essential family of transmembrane proteins, widely participate in signal transduction in organisms and have long been recognized as a major class of therapeutic targets. In structure-based drug design, high-resolution structures of GPCRs in both active and inactive states are essential for designing agonists and antagonists, respectively. However, obtaining experimental GPCR structures is costly, while homology modeling and artificial-intelligence-driven approaches including AlphaFold 2 and AlphaFold 3 often show reduced accuracy for active-state conformations. To address the above limitations, we propose PromptGPCR, an AlphaFold-based inference framework aiming to predict highly accurate structures of GPCRs in both active and inactive states. We provide AlphaFold-Multimer and AlphaFold 3 with biological sequences based on knowledge of structural biology as prompts to guide the models in the multi-state prediction task. Experimental results demonstrate that PromptGPCR can accurately predict active and inactive structures compared to baselines, suggesting its ability to provide structural hypotheses where state-resolved experimental structures are unavailable. Furthermore, PromptGPCR exhibits higher success rates in molecular docking than baselines, and this indicates that the predictions of PromptGPCR may have a certain degree of usability in downstream application scenarios involving specific GPCR conformational states.

## Introduction

A comprehensive assessment of G protein-coupled receptor (GPCR) structures is essential for understanding the activation mechanism and how these receptors are involved in human physiology and disease. In general, human GPCRs can be divided into 6 classes: class A (rhodopsin), class B1 (secretin), class B2 (adhesion), class C (glutamate), class F (frizzled), and class T (taste 2) [1,2]. The vast majority of GPCRs belong to class A. More than a third of marketed drugs are designed to target GPCRs, some of which are agonists that induce the transformation of GPCR structures to active states and some of which are antagonists that induce the transformation of GPCR structures to inactive states. For GPCR structure-based drug discovery, the development of agonists and antagonists requires highly accurate active and inactive states, respectively [3]. However, resolving GPCR structures using wet-lab experimental methods such as x-ray crystallography [4] and cryo-electron microscopy [5,6] is time-consuming and costly. There are about 800 members of human GPCRs [7], but as of 2024 December 31, only 222 human GPCRs have experimental structures in GPCRdb [8], and 149 of these GPCRs have only an active or an inactive state. Therefore, the development of highly efficient and accurate GPCR multi-state structure prediction methods is of great value in structural biology and drug discovery.

In order to facilitate the resolution of these issues, researchers have been developing computational modeling methods for GPCRs, and these approaches can be categorized into homology modeling and artificial-intelligence-driven protein structure prediction models. The application of homology modeling methods, such as GPCR-I-TASSER [9], to GPCRs is limited because these methods require a sequence identity greater than a threshold (usually 30% [10]) between target sequences and sequences of homologous templates. In recent years, artificial-intelligence-driven protein structure prediction methods have substantially improved, most notably with AlphaFold 2 [11] developed by Google DeepMind. Nevertheless, AlphaFold 2 has limitations in predicting GPCR multi-state structures. For the majority class A GPCRs, the predicted conformations tend to resemble more closely the inactive states. The emergence of AlphaFold 3 extends prediction to biomolecular complexes and indicates that deep learning models can help researchers to explore the underlying mechanisms of biological activities. AlphaFold 3 [12] outperforms AlphaFold 2 in the structural prediction of GPCRs, exhibiting enhanced accuracy in overall GPCR backbone prediction [13]. However, AlphaFold 3 cannot solve the multi-state prediction problem for GPCRs directly.

To leverage the potential of AlphaFold for GPCR multi-state structure prediction, researchers have attempted to alter the model input, including multiple sequence alignments (MSAs) and structure templates. Del Alamo *et al.* [14] used shallow MSA to guide AlphaFold 2 to predict specific GPCR conformations. By introducing multisite-directed mutations into MSA, Stein and Mchaourab [15] tried to use AlphaFold 2 to generate diverse conformations. Wayment-Steele *et al.* [16] steered AlphaFold 2 to predict alternative structures by clustering MSAs based on sequence similarity. However, these MSA optimization-based methods can only induce AlphaFold 2 to predict different conformations but cannot generate GPCR state-specific conformations. In addition, some methods aim to optimize the input templates to guide the structure predictions of GPCRs. For example, AlphaFold-MultiState [17], an extension of AlphaFold 2, omits MSA information and relies solely on active- and inactive-state templates annotated in the GPCRdb to predict specific conformational states. AlphaFold-MultiState can also predict various conformations of GPCRs or kinases by searching structure template databases online and down-sampling the MSA. However, template-based methods rely on high-quality protein structural templates and might output low-accuracy prediction results when proper state-specific templates are not accessible.

In this paper, we propose PromptGPCR, a framework based on AlphaFold and state-specific protein sequence prompts. Our experimental results show that PromptGPCR can accurately predict the active and inactive states of GPCR structures, particularly at the ligand-binding site. It evidently outperforms the baselines in protein backbone structure prediction and can predict GPCR conformations that show an evident tendency to the specific state. Moreover, our molecular docking experiments show that PromptGPCR achieves docking success rates better than baselines. It is noted that the strategy of incorporating G proteins into prompts to guide AlphaFold in predicting state-specific structures has garnered much attention from other researchers. For instance, Cheng *et al.* [18] proposed a *de novo* design pipeline for GPCR exoframe modulators, in which G proteins are introduced as structural prompts to direct the model toward predicting the active-state conformations of GPCRs. However, PromptGPCR is distinct from the approach of the pipeline in both objective and application. First, whereas the pipeline used G protein prompt sequences in GPCR binder design to stabilize active-state receptor models during the refolding of designed sequences, PromptGPCR is an inference-stage framework for GPCR conformational-state modeling. Accordingly, the 2 studies differ substantially in their primary research objectives. Second, although G protein sequences were directly used to support active-state structure prediction in the protein design pipeline, the effectiveness of this strategy was not systematically evaluated. PromptGPCR provides an extensive assessment of this strategy and further validates its effectiveness through multiple experiments.

## Materials and Methods

### Overview of PromptGPCR

**Fig. 1.**
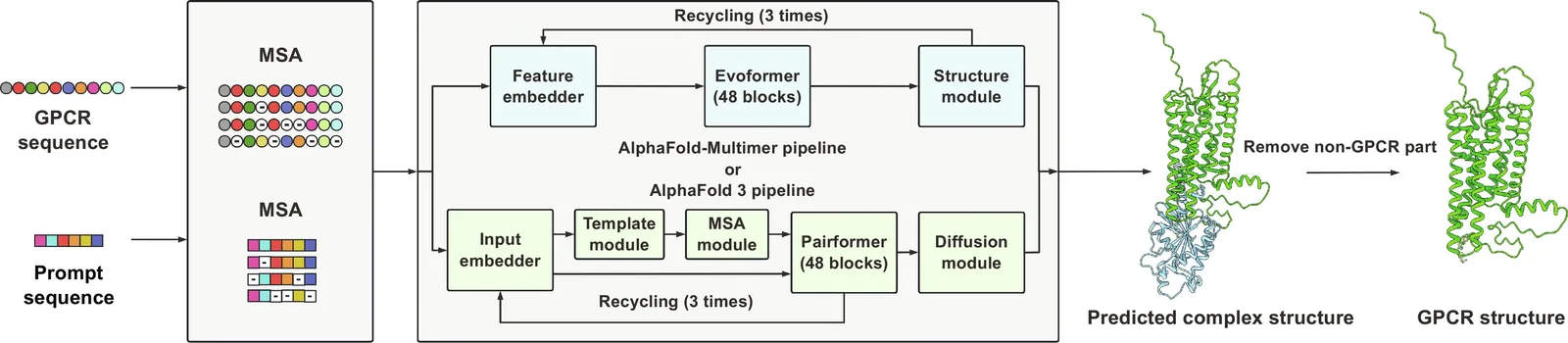
The overall framework of PromptGPCR. GPCR, G protein-coupled receptor; MSA, multiple sequence alignment.

PromptGPCR uses AlphaFold-Multimer v2.2.3 [19] and AlphaFold 3 as the basis to predict GPCR structures in specific states. Compared to AlphaFold 2 and other models, PromptGPCR introduces the concept of prompt learning to GPCR multi-state structure prediction. Amino acid sequence prompts are selected based on structural biology knowledge to enrich the contextual information of the target sequence and guide the model to perform state-specific structure prediction tasks. PromptGPCR treats the prompt sequence as an independent single chain, which is input with the target sequence into the model to predict the corresponding complex structure. The predicted structure of the target sequence represents its specified state (Fig. 1).

For the input data, PromptGPCR only supports protein sequences and MSAs and does not employ any structural templates. Consequently, the structures predicted by PromptGPCR are not guided or biased by any structural template information. In our evaluation experiments, we employed different approaches for assessing predicted structures between AlphaFold-Multimer and AlphaFold 3. For AlphaFold-Multimer, we selected the prediction with the highest confidence score from the prediction results corresponding to each target. For AlphaFold 3, considering that the prediction performance could be influenced by the random seeds and the number of structures generated per seed, we set the number of random seeds to 5 and generated 5 structures per seed, resulting in a total of 25 predicted structures for each target. Among these, only the structure with the highest ranking score was selected for all evaluation procedures.

### Data processing

To explore the performance of all methods on GPCR multi-state structures, we built a benchmark dataset. Regarding structural data, we downloaded all available structures of 113 class A GPCRs from the Protein Data Bank (PDB) [20], which were released before 2022 July 28. All PDB identifiers are from GPCRdb. Subsequently, we excluded structures whose reported resolutions are worse than 3.5 Å and all intermediate states. Finally, the benchmark dataset contains 411 structures from 107 receptors, of which 33 receptors have both active- and inactive-state structures. In addition, there are 36 and 38 GPCRs that have only inactive and active states, respectively.

To further observe the performance of all methods on PDB structures that all models did not use in their training processes, we downloaded class A GPCR structures from GPCRdb, released from 2022 July 28 to 2023 March 29. To prevent data leakage, we excluded GPCRs that have appeared in the benchmark dataset and retained only 21 newly released GPCRs, comprising 47 structures, as the PromptGPCR unseen dataset. These GPCR structures included only 3 inactive-state structures, whereas the remaining structures are active states obtained by cryo-electron microscopy. Since existing methods primarily struggle with accurately predicting active states, we removed the 3 inactive-state structures. Thus, the unseen dataset finally consisted of 44 active states of 20 GPCRs.

For sequence data, we collected the wild-type sequences of all receptors in the benchmark dataset and the unseen dataset from UniProt [21] in July 2023. Subsequently, we performed a sequence similarity analysis between the benchmark dataset and the unseen dataset. Except for GPR21 and MRGPRX1, the sequence similarity between the benchmark dataset and the unseen dataset is below 70%. Following AlphaFold-Multimer’s data pipeline, we searched for MSAs of these collected sequences. Furthermore, we aligned the wild-type sequences of the receptors with each sequence of their corresponding PDB structures. During experimental analysis, we ignored the unaligned regions of the structures as they typically represent auxiliary components or G proteins fused with GPCRs. Only GPCR-specific domains were used to calculate evaluation metrics.

### Knowledge-guided biological prompts

In PromptGPCR, the term “prompt” specifically refers to a protein sequence used to guide AlphaFold toward predicting GPCR state-specific conformations. In practice, the prompt sequence is provided to the model together with the GPCR sequence, such that AlphaFold treats the 2 input sequences as a protein complex and performs biomolecular interaction modeling. After the complex structure is predicted, the GPCR subunit within the complex can exhibit a conformation corresponding to a specific state.

We considered 2 motivations for introducing the operation of prompting on AlphaFold.

• Structural biology mechanisms of GPCRs: In the field of GPCR structural biology, when determining inactive-state GPCR structures by x-ray diffraction, researchers usually replace part of the third intracellular loop of the GPCR with the protein sequence of T4 lysozyme, which facilitates crystallization. With respect to G proteins, canonical GPCR signal transduction relies on receptor-mediated activation of heterotrimeric G proteins. In the inactive state, the G protein exists as a heterotrimer, with the Gα subunit bound to guanosine diphosphate. GPCR structural biologists have used both G proteins and T4 lysozyme as auxiliary components to resolve GPCR structures in specific functional states. Once deposited in the PDB, these structures are likely to be collected in the training datasets of AlphaFold. Hence, it is reasonable to speculate that GPCR active- and inactive-state conformations are not inherently unlearnable for AlphaFold. During the various training stages, the model may have encountered these conformational states primarily in the context of protein complexes. Consequently, enforcing complex prediction when modeling state-specific GPCR structures may help to better activate the model’s learned representations of distinct GPCR conformations.
• Role of prompts in language models: For language models, particularly large language models (LLMs) with massive trainable parameters, it is generally understood that pretraining enables them to learn rich linguistic patterns and semantic structures. Prompts can influence the conditional probability distribution of model outputs by influencing the context provided to the model. In other words, prompts serve as instructions that guide the model to follow specific patterns or perform designated tasks, which constitutes the core idea of prompt engineering in LLMs.

We argue that prompt-based guidance may also be applicable to AlphaFold-Multimer, which is built upon the Evoformer architecture, and to AlphaFold 3, which adopts the Pairformer architecture, as both can be regarded as language models trained on biological data. In biological scenarios, language models do not operate solely on sequence data, and structure data are also incorporated into the training and inference processes in the form of bias added to the attention scores. AlphaFold is trained on large-scale protein sequence and structure datasets, during which it extensively learns biomolecular interaction patterns. Therefore, introducing biologically meaningful prompt sequences to activate contextual representations of AlphaFold associated with GPCR active or inactive states is a strategy worth exploring. We believe that the key to eliciting such context lies in integrating GPCR structural biology knowledge and techniques to introduce specific protein sequences that may allow the model to recapitulate patterns encountered during its training process. Brown *et al.* [22] demonstrated that prompts can be used to reformulate various natural language processing tasks into a unified text completion problem. Pretrained LLMs learn sufficient information relevant to various downstream tasks and can be guided by prompts to perform these tasks, respectively. In the case of protein structure prediction, Hu *et al.* [23] showed that the Evoformer module in AlphaFold 2 can also function as a protein language model. Inspired by these previous works, we selected appropriate protein sequences as prompts based on structural biology knowledge of GPCRs. This may allow AlphaFold-Multimer and AlphaFold 3 to leverage domain-specific knowledge during the prediction process, guiding them toward more accurate predictions of the desired structural state.

In crystallographic experiments, the mini-Gs protein as a modified version of the G protein is often used as a substitute for the full G protein due to its high stability, small molecular weight, and its stability and ability to stabilize active receptor conformations As a result, active states of GPCRs frequently appear with the mini-Gs protein in resolved GPCR complexes [24]. Furthermore, the T4 lysozyme protein is often used as a fusion protein to enhance the stability and solubility of inactive states so that the T4 lysozyme is commonly seen with inactive states. Thus, we selected the mini-Gs and the T4 lysozyme protein as prompts to guide the model to predict active and inactive states, respectively.

### Protein–ligand docking

To further investigate the performance of PromptGPCR in potential application scenarios, we selected 9 GPCRs with active-state structures containing small-molecule ligands, from the unseen dataset as our protein–ligand docking test set. For the docking tool, we employed CSAlign-Dock [25], which combines structure alignment algorithms with docking algorithms for protein–ligand docking. CSAlign-Dock is an alignment-based protein–ligand docking approach that exploits the structural information of a known protein–ligand complex to guide the docking of a new ligand. Instead of performing blind *ab initio* docking, CSAlign-Dock first flexibly aligns the query ligand to a reference ligand extracted from an experimentally determined complex structure, thereby constraining the conformational search to the vicinity of a known binding mode. This alignment considers full ligand flexibility, including torsional degrees of freedom and ring conformations, and effectively reduces the size of the docking search space.

First, in the molecular docking experiment, we align the predicted structures with the experimental structures. Global structural alignment between the predicted structures and the experimental structures is conducted to place both protein models into a unified 3-dimensional coordinate system. This step is essential because only after structural alignment can the ligand poses generated by molecular docking be directly compared with the cocrystallized ligand poses for calculating ligand root mean square deviation (RMSD), which is a prerequisite for accurate RMSD evaluation. Then, we copy the reference ligands from the experimental structures into the predicted structures. The reason why the experiment ligands are duplicated is that CSAlign-Dock requires a reference ligand pose as input to guide the docking process. Notably, this ligand duplication procedure is consistently applied to the prediction results of PromptGPCR as well as to all baseline methods. Finally, both the ligand and the predicted structure complexes are input into CSAlign-Dock for docking.

To evaluate the docking results, we consider only the top 5 generated ligand structures ranked by energy. The RMSD was calculated between the docked ligands and the reference ligands. If at least 1 of the top 5 ligand structures has an RMSD below 2 Å, the docking result is considered successful.

## Results and Discussion

### Multi-state structure prediction via prompts

**Fig. 2.**
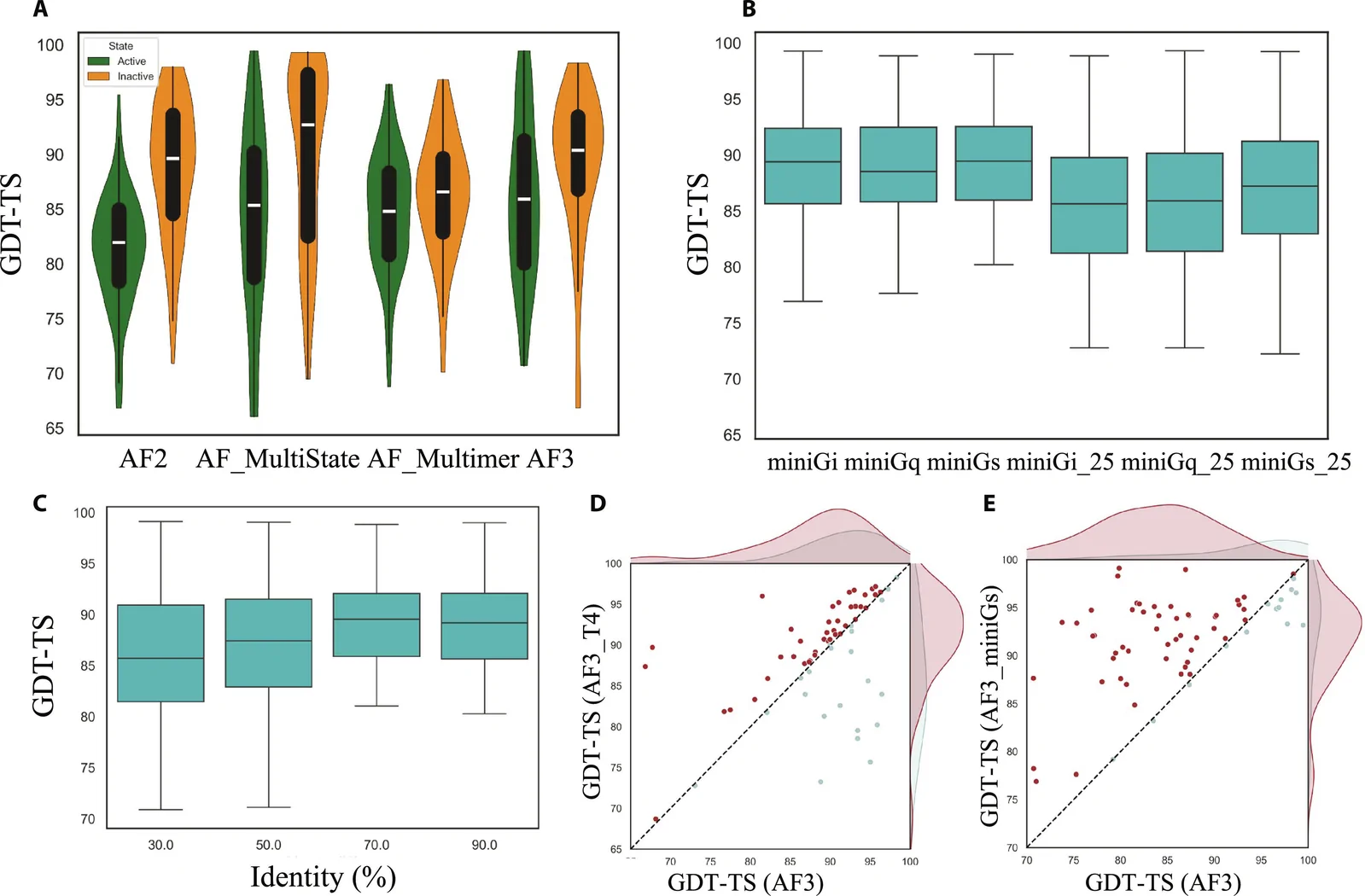
The backbone prediction accuracy of G protein-coupled receptors (GPCRs) on the PromptGPCR benchmark dataset. (A) The single-chain prediction results of the 4 models. Green represents active states, while orange represents inactive states. (B) The performance of AlphaFold-Multimer using different mini-G proteins and their truncated sequences as prompts for active-state predictions. (C) The performance of AlphaFold-Multimer using random sequences with different similarities to mini-Gs protein as prompts for active-state predictions. (D) Comparison of prediction accuracy between AlphaFold 3 with T4 prompt and AlphaFold 3 without any prompt. (E) Comparison of prediction accuracy between AlphaFold 3 with mini-Gs prompt and AlphaFold 3. Each point in (D) and (E) represents a GPCR structure whose coordinates indicate the global distance test–total score (GDT-TS) of structures predicted by AlphaFold 3 and the PromptGPCR framework based on AlphaFold 3, respectively. Red indicates that PromptGPCR predictions are better, while blue indicates the opposite.

We first assessed the performance of existing deep learning methods on GPCR multi-state structure prediction. AlphaFold 2, AlphaFold-MultiState, AlphaFold-Multimer, and AlphaFold 3 were run to predict the single-chain GPCR structures in the PromptGPCR benchmark dataset. To evaluate the performance of these models, the global distance test–total score (GDT-TS) [26] values for the predicted structures were calculated. The results of the 4 methods are shown in Fig. 2A.

For class A GPCRs, all 4 methods performed better in predicting inactive states than active states. AlphaFold-MultiState showed the best performance for inactive states with an average GDT-TS of 90.20, while AlphaFold 3 performed best in active-state predictions with an average GDT-TS of 85.76. AlphaFold 2 showed the worst performance in the active-state predictions while performing the third best in the inactive-state predictions. The average GDT-TS scores of AlphaFold 2 for inactive states were 7.47 higher than those for active states, indicating that AlphaFold 2 has a strong bias toward predicting inactive states. This bias may be caused by the fact that most class A GPCR structures in AlphaFold 2 training data are close to inactive states [17]. Consequently, the model primarily learned the mapping between sequences and inactive states. In contrast, while AlphaFold-Multimer performed the worst in predicting inactive states, the average GDT-TS for active states of AlphaFold-Multimer was 3.49 higher than that of AlphaFold 2. Furthermore, the average GDT-TS values for inactive states predicted by AlphaFold-Multimer and AlphaFold 3 are only 1.45 and 3.21 higher than those for active states, respectively, showing that AlphaFold-Multimer and AlphaFold 3 have less bias toward either state than AlphaFold 2. Since most of the complexes in GPCRdb include GPCR active-state conformations, we speculate that predictors of complex structures such as AlphaFold-Multimer and AlphaFold 3 may be trained on more GPCR active states so that they have learned more patterns associated with active states compared to AlphaFold 2.

To explore the space of active states of the GPCRs learned by AlphaFold-Multimer and AlphaFold 3 during training, we used appropriate protein sequence prompts to guide the model to predict active states. First, we tried 3 kinds of mini-G proteins—mini-Gs, mini-Gi, and mini-Gq—and the first 25 amino acids near the C-terminal region of these mini-G proteins as prompts to predict the GPCR active states by using AlphaFold-Multimer. As shown in Fig. 2B, all 3 mini-G proteins can improve prediction accuracy, with average GDT-TS scores above 88, outperforming single-chain predictions from AlphaFold-Multimer. Among them, mini-Gs prompted the highest average GDT-TS and the most consistent results, which showed slightly better performance than other mini-G proteins. Furthermore, truncated mini-G proteins did not perform as well as full-length mini-G proteins, indicating that shorter prompts are less effective.

To verify the effectiveness of the sequence prompts selected based on biological knowledge, we randomly generated sequences with varying similarity to the mini-Gs protein and used these partially random sequences as prompts to predict the active states. As shown in Fig. 2C, the accuracy of the prediction improved as the sequence similarity increased. Therefore, the mini-Gs protein sequence helps capture the content of active states and was used to predict active states in subsequent experiments. Then, we used the T4 lysozyme as the prompt to predict inactive states. Compared to the single-chain prediction of AlphaFold-Multimer, the T4 lysozyme increased the average GDT-TS score by 1.85, which was very close to that of AlphaFold 2. As such, the T4 lysozyme was chosen as the prompt for inactive states.

To further clarify the promoting effect of T4 lysozyme on inactive-state structure prediction, we attempted to introduce a negative control for it. We selected dihydrofolate reductase (UniProt accession: P0ABQ4) as the negative control for the inactive-state prompt. This sequence was selected because, compared with the T4 lysozyme sequence, it has a similar sequence length but a sequence identity lower than 25%, and it is a nonhuman sequence. We believe that there is currently no evidence showing that this sequence can meet the requirements for preparing fusion proteins. Therefore, this sequence can be considered unrelated to both GPCR inactive-state structures and the T4 lysozyme sequence. As shown in Fig. A3, the 2 inactive-state prompt sequences produced highly similar modeling effects, and the average GDT-TS scores between the predicted results and the inactive-state GPCR structures were both higher than 90. This suggests that both sequences may help PromptGPCR improve its performance in inactive-state structure prediction, although the control sequence selected in this study currently has no real association with GPCRs. We do not consider this to indicate that the T4 lysozyme is completely nonspecific for inactive-state modeling by PromptGPCR; however, whether this promoting effect may also exist for more prompt sequences remains to be further validated.

Furthermore, to further present the prediction results of PromptGPCR, we compared the predictions generated by PromptGPCR in active- or inactive-state structure prediction scenarios with the corresponding experimental structures. As shown in Fig. A2, for the 2 GPCRs examined, the active- and inactive-state predictions generated by PromptGPCR exhibit clear overall differences, particularly in the sixth transmembrane helix region. In addition, compared with the inactive-state predictions, the active-state predictions are closer to the active-state experimental structures. The opposite pattern can be observed in the comparison with the inactive-state experimental structures.

To evaluate the effectiveness of the prompts, these 2 prompts were also utilized in AlphaFold 3. Figure 2D and E demonstrate that incorporating the T4 lysozyme and mini-Gs as prompts improves the performance of AlphaFold 3 in predicting the inactive states and active states, respectively. For inactive-state prediction, AlphaFold 3 with the T4 lysozyme prompt outperforms AlphaFold 3 without a prompt in 44 out of 67 targets. Similarly, for active-state prediction, AlphaFold 3 with the mini-Gs prompt performs better than without a prompt on 52 out of 66 targets.

**Fig. 3.**
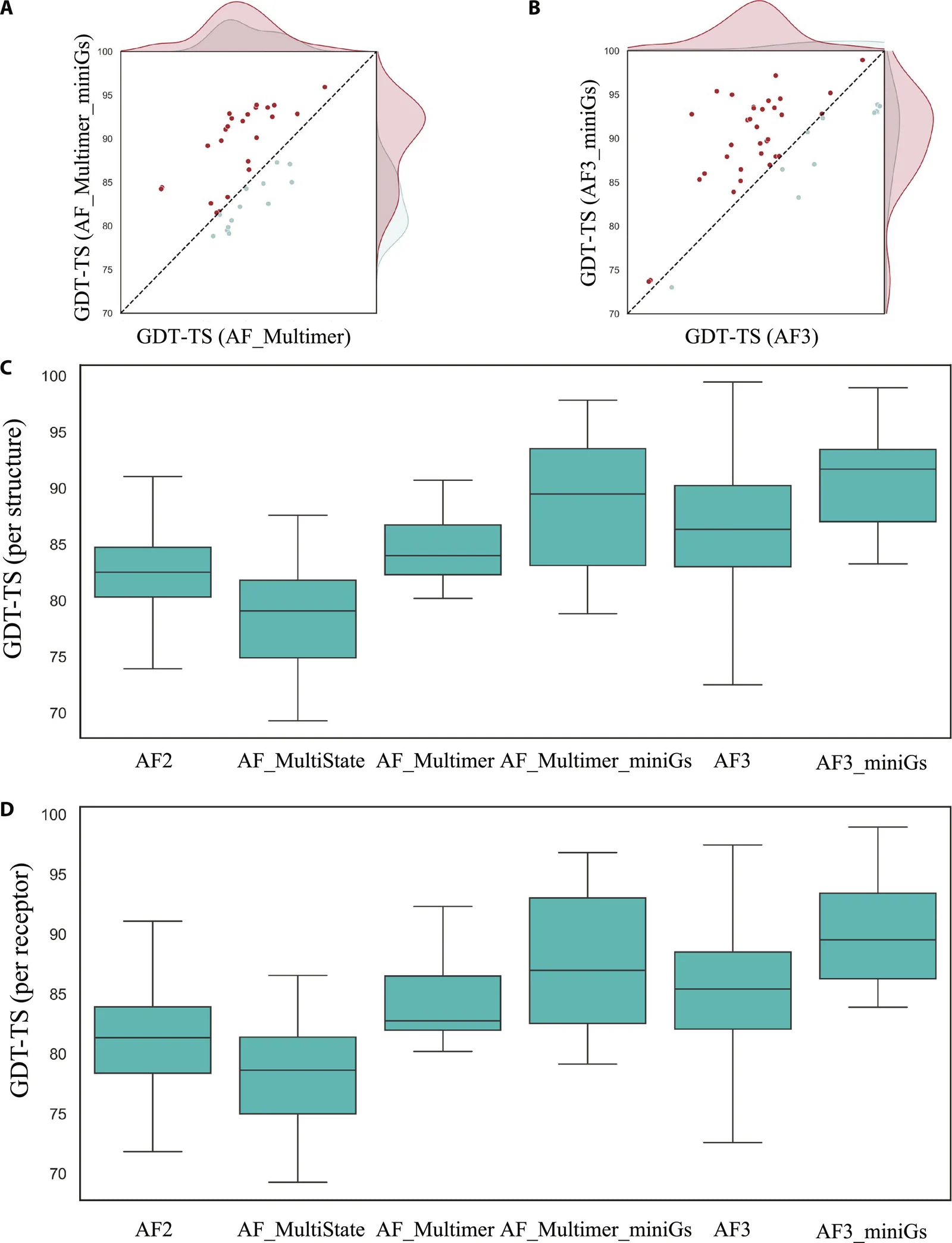
Prediction accuracy of G protein-coupled receptors (GPCRs) on the unseen PromptGPCR dataset. (A) Active-state prediction accuracy: AlphaFold-Multimer with *vs.* without the mini-Gs prompt. (B) Prediction accuracy: AlphaFold 3 with *vs.* without the mini-Gs prompt. Each dot in (A) and (B) corresponds to one GPCR structure; axes denote global distance test–total score (GDT-TS) values from baseline models and PromptGPCR. Red dots denote superior PromptGPCR predictions, and blue dots denote the reverse. (C and D) Prediction accuracy of AlphaFold 2, AlphaFold-MultiState, AlphaFold-Multimer, mini-Gs prompt-augmented AlphaFold-Multimer, AlphaFold 3, and mini-Gs prompt-augmented AlphaFold 3 on unseen data. Results are presented (C) per structure and (D) per receptor.

However, since we collected GPCR structures before 2022 July 28, a large proportion of structures in the PromptGPCR benchmark dataset may also be included in the training data of AlphaFold 2, AlphaFold-Multimer, and AlphaFold 3. To evaluate the generalizability of PromptGPCR, we compared PromptGPCR with other methods on the unseen dataset. As shown in Fig. 3C, the average GDT-TS of AlphaFold 3 with a mini-Gs prompt was 89.76, which is notably higher than those of AlphaFold-Multimer (GDT-TS = 84.24), AlphaFold 2 (GDT-TS = 81.47), and AlphaFold-MultiState (GDT-TS = 78.22). The per-receptor evaluation in Fig. 3D yields identical findings. We hypothesize that AlphaFold-MultiState performed worse than AlphaFold 2 mainly because the GPCR structures in the unseen dataset are newly released structures, which cannot find templates with a high sequence identity. We also performed 2 pairwise comparisons between the models with and without a prompt. As shown in Fig. 3A and B, AlphaFold-Multimer with a mini-Gs prompt outperformed AlphaFold-Multimer on 31 out of 44 targets. AlphaFold 3 with a mini-Gs prompt outperformed AlphaFold 3 on 33 out of 44 targets. This demonstrates that the framework of prompting can consistently help different multimer structure prediction models to better predict GPCR multi-state conformations.

### Application of high-precision multi-state GPCR conformations

**Fig. 4.**
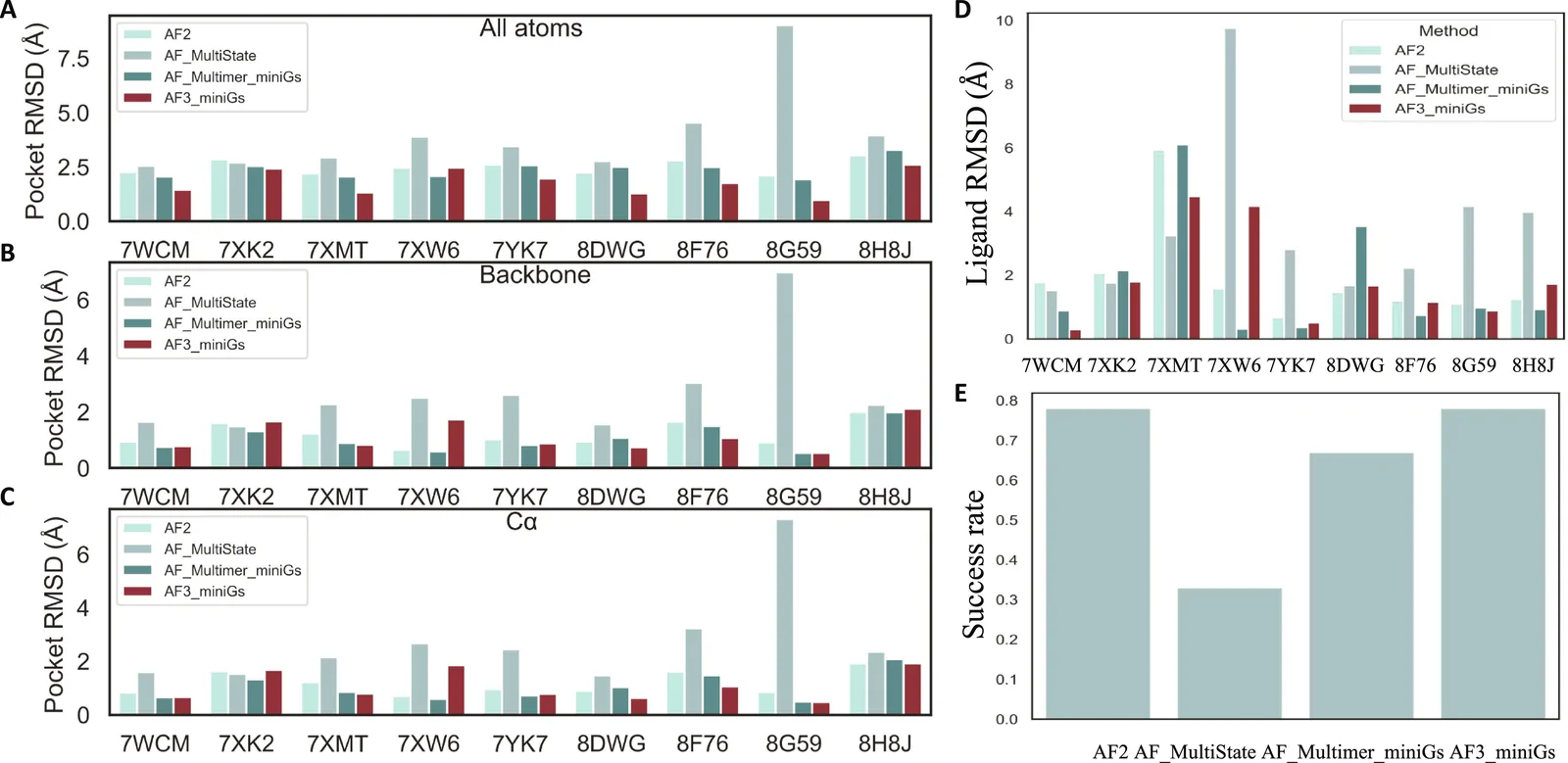
Prediction performance of 4 models on the docking test set. The all-atom root mean square deviation (RMSD) (A), backbone RMSD (B), and Cα RMSD (C) of structures predicted by AlphaFold 2, AlphaFold-MultiState, AlphaFold-Multimer with mini-Gs, and AlphaFold 3 with mini-Gs. (D) The docking results of experimental structures and different predicted structures. Ligand RMSD is the minimum value of RMSD between the top 5 ligand poses generated by CSAlign-Dock and the experimental ligand pose. (E) The success rate of docking experiments for different predicted structures. If the ligand RMSD is less than 2 Å, it is counted as a successful docking.

One key application of high-quality GPCR modeling is to obtain the protein–ligand complex, which is helpful for virtual screening in drug design. To explore the potential application of PromptGPCR in drug design, we calculated the pocket region RMSD of the structures predicted by AlphaFold 3 with a prompt, AlphaFold-Multimer with a prompt, AlphaFold 2, and AlphaFold-MultiState on the docking test set. Because the pocket region is the binding site between a receptor and a ligand, the prediction accuracy of the pocket region affects the binding affinity of a predicted structure to the ligand, which is crucial for the design of GPCR-targeted drugs. Figure 4A, B, and C respectively show the full-atom, backbone-atom, and Cα-atom RMSDs for the 4 methods in the pocket region, which show that AlphaFold 3 with a prompt outperformed the other methods in predicting the pocket region.

**Fig. 5.**
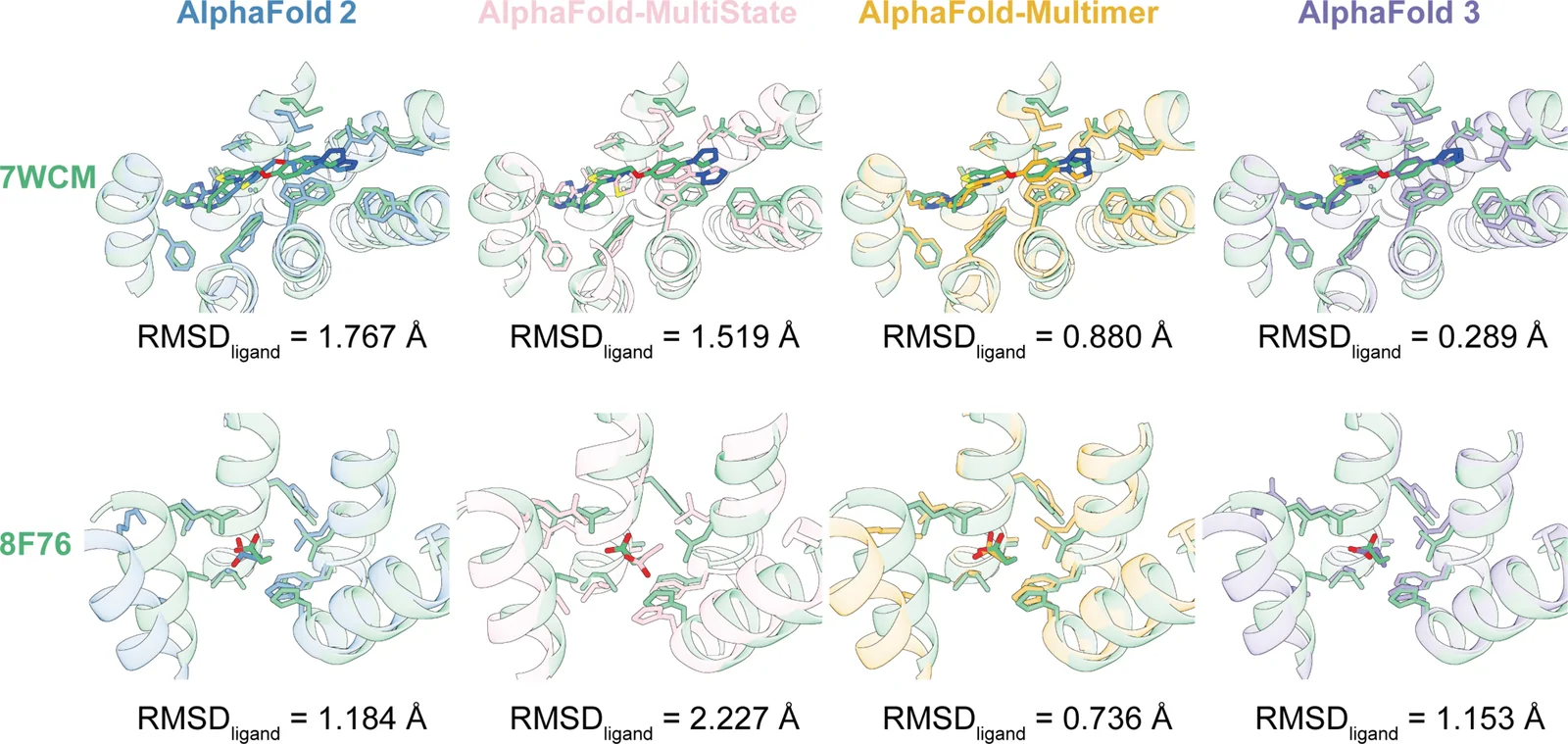
Docking results of structures predicted by AlphaFold 2, AlphaFold-MultiState, AlphaFold-Multimer, and AlphaFold 3 with a prompt (PDB IDs: 7WCM and 8F76). Green denotes the experimental structures. RMSD, root mean square deviation.

Additionally, we utilized the CSAlign-Dock tool to perform docking experiments on the docking test set. As shown in Fig. 4D and E, among the 4 structure prediction methods, AlphaFold-MultiState performed the worst, with an average ligand RMSD of 3.46 Å and a docking success rate of only 33.33%. This result is consistent with the poor performance of AlphaFold-MultiState in the prediction of the pocket region, indicating that inaccurate predictions are not suitable for docking. AlphaFold 3 with mini-Gs achieved an average ligand RMSD of 1.85 Å and a docking success rate of 77.78%, outperforming all other methods. Figure 5 shows the docking results for 2 GPCRs predicted by AlphaFold 3 with mini-Gs, AlphaFold-Multimer with mini-Gs, AlphaFold 2, and AlphaFold-MultiState. As shown in the figure, the pocket region of the 7WCM and 8F76 structures predicted by AlphaFold 3 with mini-Gs exhibits slight differences compared to the experimental structures, which are better than the structures predicted by other methods. Additionally, in the prediction of AlphaFold 3 with a prompt, the docked ligands are also closely similar to the experimental ligands in terms of both locations and poses. Overall, the docking success rates of the structures predicted by PromptGPCR were closer to those of the experimental structures than those of the other 2 methods, suggesting that PromptGPCR can lead to more accurate predicted structures for docking. However, it should be clarified that the molecular docking experiments in this study are essentially guided pose-recovery tests, which differ from scenarios such as blind docking. The objective of our experiments was to investigate whether the predicted GPCR state-specific structures could recover binding modes that are closer to those observed in the true cocrystal structures during molecular docking. This experimental setting indeed differs from the practical requirements of scenarios such as virtual screening. Therefore, the results of the molecular docking experiments do not support the conclusion that PromptGPCR can achieve excellent performance in virtual screening scenarios.

## Conclusions

As the largest family of transmembrane proteins, GPCRs have active and inactive states and participate in many cellular activities through transitions between the 2 states. Thus, GPCRs are highly popular drug targets. The multi-state structure prediction of GPCRs is valuable for both drug design and biological research. Existing multi-state prediction methods either fail to accurately predict structures in specific states or rely on high-quality template structures. Thus, we propose a strategy based on biological sequence prompts, called PromptGPCR. PromptGPCR selects 2 protein sequences as prompts: mini-Gs for active states and the T4 lysozyme for inactive states.

Our experimental results show that PromptGPCR can accurately predict the specified GPCR states, outperforming the baseline models in backbone structure prediction. Moreover, PromptGPCR based on AlphaFold 3 achieves the highest docking success rate, closely approximating the experimental structures.

We expect that PromptGPCR can provide a certain degree of support for downstream applications involving specific GPCR conformational states. However, PromptGPCR still exhibits several limitations regarding the computational modeling of GPCRs in specific states.

First, as integral transmembrane proteins, lipids play a fundamental role in the folding and conformational stability of membrane proteins. For traditional computational methods, such as molecular dynamics simulations, the existence of lipids is a critical prerequisite for ensuring simulation validity and stable convergence. In the context of deep-learning-based protein structure prediction models, such as AlphaFold-Multimer or AlphaFold 3, prediction accuracy relies heavily on the training process using experimental structures from the PDB. Due to inherent constraints in modeling capacity and the need for efficient computational resource allocation, auxiliary molecules, such as lipid molecules, which are crucial for maintaining structural integrity, are typically excluded from the training datasets and treated as heteroatoms to be removed. PromptGPCR is built upon the AlphaFold framework and cannot address these specific limitations.

Second, the relationship between G protein classes and complex structure prediction performance is a research direction to be developed. Mini-G proteins are engineered Gα subunits that stabilize specific active-state conformations of GPCRs by mimicking the coupling interface of distinct heterotrimeric G protein classes. Different G protein families (Gs, Gi/o, Gq/11, and G12/13) are known to engage GPCRs in class-specific manners, which can influence receptor conformational ensembles, downstream signaling pathways, and potentially ligand-binding poses through allosteric effects. Consequently, the choice of a particular mini-G protein may affect the predicted GPCR–ligand complex by biasing the receptor toward a specific signaling-competent state. Thus, exploring the potential link between G protein subtypes and the performance of state-specific GPCR structure prediction is warranted, as the findings would be instrumental in developing further methods.

Third, while PromptGPCR focuses primarily on GPCRs, it is possible that this method can also be applied to other protein families that have multiple conformational states. Some previous studies [27,28] have attempted to use AlphaFold for modeling other proteins with multiconformational properties, and PromptGPCR is expected to help facilitate similar studies. If appropriate protein sequence prompts can be identified, other protein families can also benefit from the PromptGPCR framework to predict multi-state structures. Note that the construction of prompts relies on prior knowledge of structural biology. A future direction is to explore the use of generative models or prompt tuning [29] to automatically generate appropriate sequence prompts. Another possible direction for improving PromptGPCR is to refine case studies. For studies that investigate the binding modes and conformational dynamics of certain GPCR members using molecular dynamics simulations and AlphaFold [30–32], PromptGPCR may provide additional support.

Fourth, olfactory receptors (ORs) are also an important class within the GPCR superfamily, and because current structural biology methods have difficulty in achieving stable success with ORs, computational modeling is of great significance for obtaining OR structures. Existing studies have investigated the performance of AlphaFold on ORs [33], whereas PromptGPCR lacks a systematic evaluation of ORs. This is a research direction that can be further explored in the future work of PromptGPCR.

Finally, the exploratory objective of PromptGPCR is to expand the capability of AlphaFold in predicting active-state and inactive-state structures. However, the current experimental results still require further strengthening to validate the effectiveness of the inactive-state prompt. Compared with the strong structural–biological association between G proteins and GPCRs, the T4 lysozyme-based fusion protein strategy may not fully meet our expectations. Nevertheless, we still consider PromptGPCR to have certain practical value for researchers focusing on inactive-state modeling. Further extending the usability of the existing framework for inactive-state structure prediction will be one of the future directions of PromptGPCR.

## Data Availability

Related data, running scripts, and other instructions are available at https://github.com/PAFFMew/PromptGPCR.

## Supplementary Materials

Supplementary 1 Figs. A1 to A3 Tables A1 to A4
