# Supplementary material for: Multi-state Structure Prediction of G Protein-Coupled Receptor Proteins via Prompting on AlphaFold
Source: Comput Struct Biotechnol J. 2026 Sep 10;35(1):0179. doi: 10.34133/csbj.0179 (PMC13558818; doi:10.34133/csbj.0179)
Supplement: Supplementary file 1 [file csbj.0179.f1.pdf]

Appendix A. Supplementary Information and Appendices

Table A.1: Active states of PromptGPCR benchmark dataset

PDB ID	Receptor
6H7O	β1_turkey
7JHJ	BILF1_strain
7E2Y	5-HT1A_Human
7E32	5-HT1D_Human
7E33	5-HT1E_Human
7EXD	5-HT1F_Human
6WHA	5-HT2A_Human
7XT8	5-HT4_Human
7UM5	5-HT5A_Human
7XTB	5-HT6_Human
7XTC	5-HT7_Human
7LD4	A1_Human
5G53	A2A_Human
6OS1	AT1_Human
7EIB	B1_Human
7F2O	B2_Human
7VKT	BLT1_Human
6PT0	CB2_Human
7MBX	CCK1_Human
7F8W	CCK2_Human
7VL9	CCR1_Human
7O7F	CCR5_Human
6WWZ	CCR6_Human
6LFO	CXCR2_Human
7JV5	D1_Human
7JVR	D2_Human
7CMV	D3_Human
7CX3	EP2_Human
7D7M	EP4_Human
7T6T	FPR1_Human

Continued on next page

Table A.1 continued from previous page

PDB ID	Receptor
6OMM	FPR2/ALX_Human
7WQ3	GAL1_Human
7WQ4	GAL2_Human
7F9Z	ghrelin_Human
7CFM	GPBA_Human
7VUG	GPR139_Human
7TUZ	GPR183_Human
7EJX	GPR88_Human
7DFL	H1_Human
7FIH	LH_Human
7TD1	LPA1_Human
6OIJ	M1_Human
7F4I	MC1_Human
7AUE	MC4_Human
7S8N	MRGPRX2_Human
7S8P	MRGPRX4_Human
7DB6	MT1_Human
7RMG	NK1_Human
6OS9	NTS1_Human
7RYC	OT_Human
7L1V	OX2_Human
3DQB	Rhodopsin_Bovine
4ZWJ	Rhodopsin_Human
7EW7	S1P1_Human
7T6B	S1P2_Human
7EW3	S1P3_Human
7EW1	S1P5_Human
7T10	SST2_Human
7KH0	V2_Human
7VGX	Y1_Human
7EJ0	α2A_Human
6K41	α2B_Human
3SN6	β2_Human
7DH5	β3_Dog

Continued on next page

Table A.1 continued from previous page

PDB ID	Receptor
6B73	κ_Human
5C1M	μ_Mouse

Table A.2: Inactive states of PromptGPCR benchmark dataset

PDB ID	Receptor
6I9K	Rhodopsin_Hasarius
2Z73	Rhodopsin_squid
2VT4	β1_turkey
7C61	5-HT1B_Human
6A94	5-HT2A_Human
6DRZ	5-HT2B_Human
6BQH	5-HT2C_Human
7UM4	5-HT5A_Human
5UEN	A1_Human
3EML	A2A_Human
4YAY	AT1_Human
5UNG	AT2_Human
7K15	BLT1_Human
5O9H	C5a1_Human
5ZTY	CB2_Human
7F8U	CCK1_Human
5T1A	CCR2_Human
4MBS	CCR5_Human
6QZH	CCR7_Human
5LWE	CCR9_Human
6LFL	CXCR2_Human
3OE9	CXCR4_Human
6RZ4	CysLT1_Human
6RZ7	CysLT2_Human
6CM4	D2_Human
3PBL	D3_Human

Continued on next page

Table A.2 continued from previous page

PDB ID	Receptor
5WIV	D4_Human
6D26	DP2_Human
5YWY	EP4_Human
5X93	ETB_Human
6KO5	ghrelin_Human
7BR3	GnRH1_Human
7TUY	GPR183_Human
3RZE	H1_Human
4Z34	LPA1_Human
5CXV	M1_Human
3UON	M2_Human
4DAJ	M3_Rat
5DSG	M4_Human
6OL9	M5_Human
6W25	MC4_Human
6E59	NK1_Human
4EA3	NOP_Human
6ZIN	NTS1_Rat
6TPK	OT_Human
4ZJ8	OX1_Human
4S0V	OX2_Human
4XNW	P2Y1_Human
4NTJ	P2Y12_Human
5ZKQ	PAF_Human
3VW7	PAR1_Human
5NDD	PAR2_Human
1F88	Rhodopsin_Bovine
3V2Y	S1P1_Human
6RNK	succinate_Rat
6IIV	TP_Human
7DDZ	Y2_Human
7B6W	α1B_Human
6KUX	α2A_Human
6KUW	α2C_Human

Continued on next page

Table A.2 continued from previous page

PDB ID	Receptor
7BVQ	β1_Human
2RH1	β2_Human
4N6H	δ_Human
4EJ4	δ_Mouse
4DJH	κ_Human
4DKL	μ_Mouse

Table A.3: Details of PromptGPCR unseen dataset

GPCR	# Structure	Highest Resolution (Å)	UniProt Accession	Representative PDB ID
CCR3	1	3.1	P51677	7X9Y
GPR17	1	3	Q13304	7Y89
GPR119	4	2.3	Q8TDV5	7XZ5
MRGPRX1	3	2.7	Q96LB2	8DWC
SST4	2	2.8	P31391	7XMS
TSH	5	2.4	P16473	7T9M
ACKR3	3	3.3	P25106	7SK4
51E2	1	3.1	Q9H255	8F76
A2B	2	2.9	P29275	8HDO
BB2	2	3	P30550	7W3Z
ETA	1	3	P25101	8HCQ
FFA4	6	2.6	Q5NUL3	8G59
FSH	1	2.8	P23945	8I2G
GPR20	2	3.1	Q99678	8HS2
GPR21	3	2.9	Q99679	8HJ2
GPR35	1	3.2	Q9HC97	8H8J
HCA2	1	3.1	Q9H255	7XK2
MC2	1	3.3	Q01718	8GY7
RXFP1	1	3.2	Q9HBX9	7TMW
RXFP4	3	2.8	Q8TDU9	7YJ4

Table A.4: Details of docking test set

GPCR	PDB ID	Resolution (Å)	Chain ID	PDB Date
GPR119	7WCM	2.3	R	2022/12/21
HCA2	7XK2	3.1	R	2023/2/22
SST4	7XMT	2.8	R	2022/8/3
TSH	7XW6	2.8	R	2022/8/17
RXFP4	7YK7	2.8	R	2023/3/1
MRGPRX1	8DWG	2.7	R	2022/11/2
51E2	8F76	3.1	A	2023/3/22
FFA4	8G59	2.6	R	2023/3/8
GPR35	8H8J	3.2	C	2023/2/8

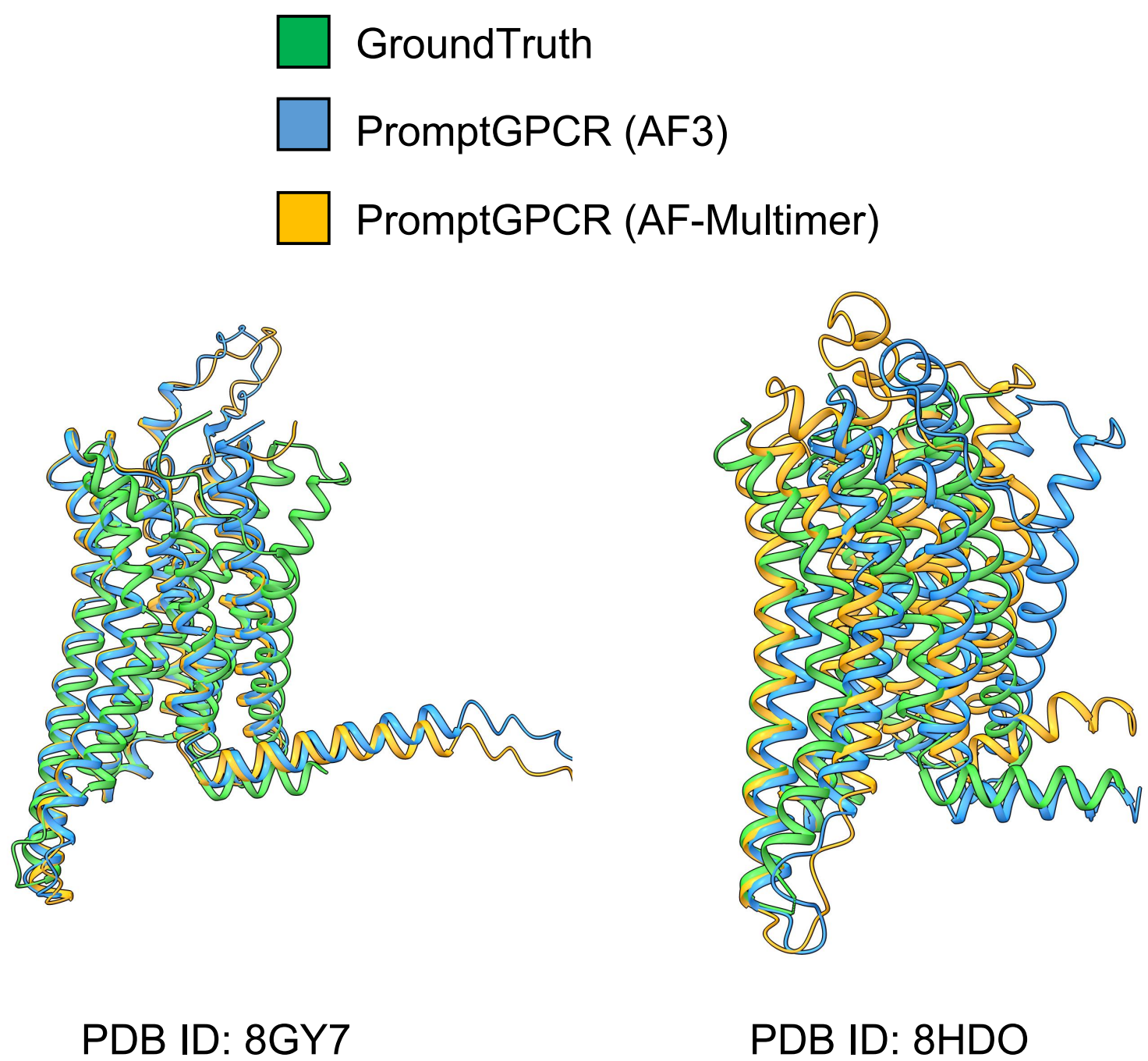

Figure A.1: Visualization of the comparison between the structures predicted by PromptGPCR and the experimental results for the unseen examples in the training set of AlphaFold-Multimer and AlphaFold 3.

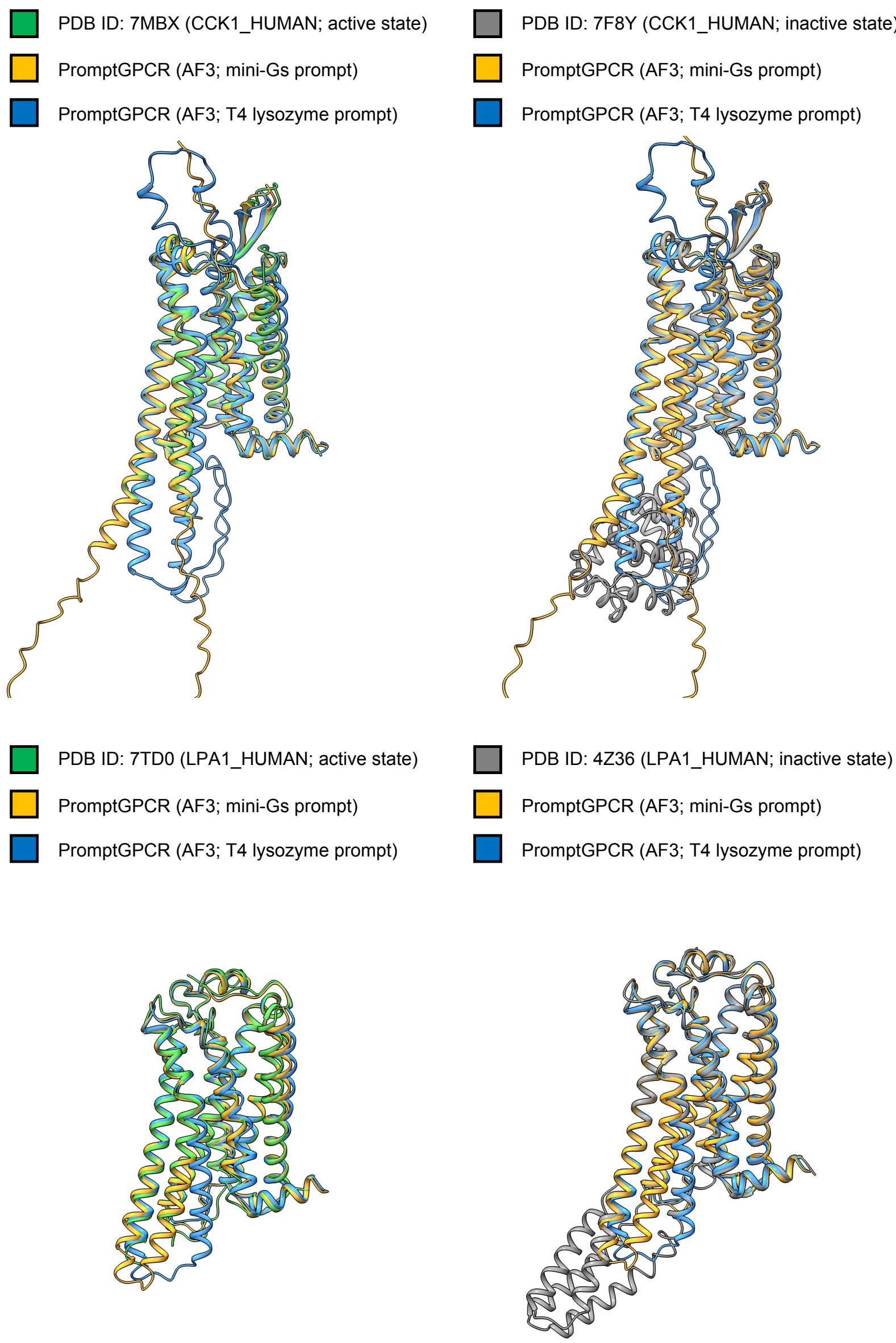

Figure A.2: Comparison of PromptGPCR predictions with various prompt sequences for receptors with experimentally resolved active and inactive states. Experimental active-state structures are shown in green, experimental inactive-state structures in grey, predictions with mini-Gs in yellow and predictions with T4 lysozyme in blue.

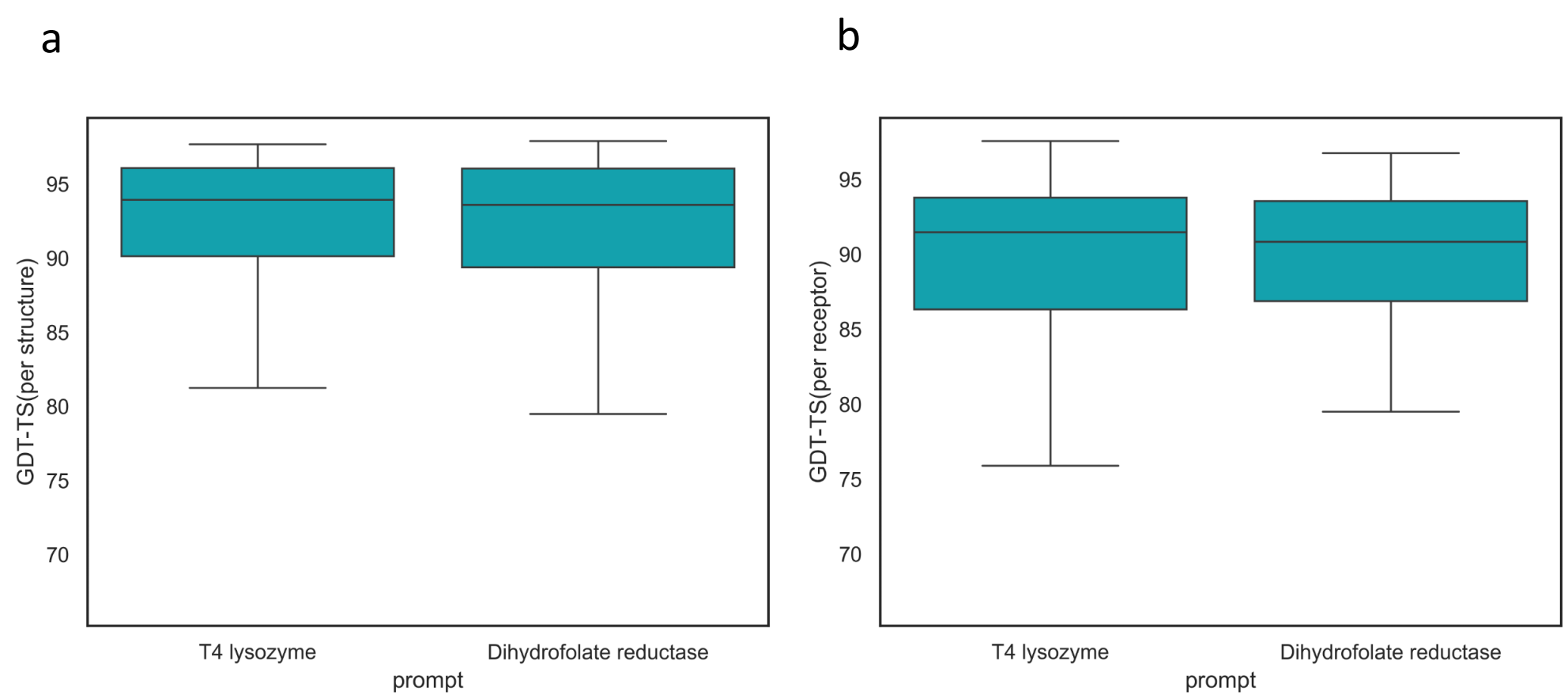

Figure A.3: Comparison of prediction accuracy of inactive states between AlphaFold3 with T4 lysozyme prompt and AlphaFold 3 with the the Dihydrofolate reductase prompt on the PromptGPCR benchmark dataset. (a) Compared by PDB structures (b) Compared by receptors.
